# A Passive Wireless Multi-Sensor SAW Technology Device and System Perspectives

**DOI:** 10.3390/s130505897

**Published:** 2013-05-10

**Authors:** Donald C. Malocha, Mark Gallagher, Brian Fisher, James Humphries, Daniel Gallagher, Nikolai Kozlovski

**Affiliations:** Electrical Engineering & Computer Science Department, University of Central Florida, Orlando, FL 32816, USA; E-Mails: mgallagher@knights.ucf.edu (M.G.); brianfisher2@gmail.com (B.F.); james.humphries@knights.ucf.edu (J.H.); dgallagher@knights.ucf.edu (D.G.); nikolaik@cfl.rr.com (N.K.)

**Keywords:** surface acoustic wave, RFID, sensor, spread spectrum

## Abstract

This paper will discuss a SAW passive, wireless multi-sensor system under development by our group for the past several years. The device focus is on orthogonal frequency coded (OFC) SAW sensors, which use both frequency diversity and pulse position reflectors to encode the device ID and will be briefly contrasted to other embodiments. A synchronous correlator transceiver is used for the hardware and post processing and correlation techniques of the received signal to extract the sensor information will be presented. Critical device and system parameters addressed include encoding, operational range, SAW device parameters, post-processing, and antenna-SAW device integration. A fully developed 915 MHz OFC SAW multi-sensor system is used to show experimental results. The system is based on a software radio approach that provides great flexibility for future enhancements and diverse sensor applications. Several different sensor types using the OFC SAW platform are shown.

## 1. Introduction

Surface acoustic wave (SAW) technology is beginning to attract serious interest for a broad range of sensor applications, especially in aerospace and health monitoring applications [1,2]. Many applications have very challenging requirements: maintenance-free (no battery), no external power (scavenging or external power source), reliable life-cycle (years in a wing structure or hours in an engine exhaust), light and small, *etc.* A short list of system specifications may include simultaneous multi-sensor interrogation and reception, wireless, passive, radiation hard, and range of several centimeters to 100s of meters. The sensors should be small, rugged, provide radio frequency identification (RFID) on chip, operate under conditions ranging from cryogenic to high temperature, and differing embodiments should provide temperature, gas pressure, strain, chemo- or bio- detection and others.

Over the last 25 years there have been several proposed SAW embodiments for wireless, passive SAW RFID sensors, which include narrowband resonant devices, reflective delay line sensors, SAW chirp devices, external-sensor-SAW module, and code division multiple access (CDMA) [2–7]. Narrowband devices can provide an ID through differing resonant frequency per device, while most delay line devices provide the coding through pulse position reflectors. The chirp sensor uses the correlation properties for enhanced sensor data extraction, but provides no effective multi-coding.

Initial work on orthogonal frequency coded (OFC) SAW devices for RFID and communication began in 2000, and the first publication on SAW OFC was in 2004 [8,9]. The implementation of OFC in a SAW structure provides the greatest flexibility in time, frequency and code diversity. This adaptability has advantages in a multi-sensor system for identification and sensor accuracy, which will be discussed. The device and systems to be discussed are based on an operational center frequency of 915 MHz and bandwidth of approximately 74 MHz. The five chip OFC reflectors are used for encoding each device on YZ LiNbO_3_ and the devices are connected to a folded dipole antenna for reception and re-transmission of the interrogation signal.

## 2. Background

There have been a number of publications on the theory and approach to OFC based on communication theory, and then its application to SAW device embodiments [8,9]. A short review follows: consider a time limited, nonzero time function defined as:

(1) $$h\left(t\right)=\;\sum_{n=0}^{N-1}a_{n}\;.\varphi_{n}\left(t\right)\;.\text{rect}\;(\frac{t}{\tau })\;\text{where}\;\varphi_{n}\left(t\right)\;=\cos\left(\frac{n\pi t}{\tau }\right)\;\text{and}\;\text{rect}\left(x\right)=\{\begin{array}{ll} 1, & \left|x\right|\leq 0.5\\ 0, & \text{otherwise} \end{array}$$

The function *φ_n_(t)*, represents a complete orthogonal basis set with real coefficients *0* ≤ *a_n_* ≤ *1*. The members of the basis set are orthogonal over the given time interval if:

(2) $$\int_{-\frac{\tau }{2}}^{\frac{\tau }{2}}\varphi_{n}\left(t\right)\;\cdot \;\varphi_{m}\left(t\right)dt=\{\begin{array}{ll} K_{n,} & n=m\\ 0, & n\neq m \end{array}$$

Given the basis set and constraints, two functional descriptions are obtained which have the forms:

(3) $$h_{1}\left(t\right)=\sum_{n=0}^{N}a_{n}\;\cdot \;\cos\;\left(\frac{2n\;\cdot \;\pi t}{\tau }\right)\;\cdot \;\text{rect}\left(\frac{t}{\tau }\right)$$

(4) $$h_{2}\left(t\right)=\sum_{m=0}^{M}b_{m}\;\cdot \;\cos\left(\frac{\left(2m+1\right)\;\cdot \;\pi t}{\tau }\right)\;\cdot \;\text{rect}\left(\frac{t}{\tau }\right)$$

Each cosine term in the summations in Equations (3) and (4) represent a time-gated sinusoid whose local center frequencies are given by:

(5) $$f_{n}=\frac{t}{\tau }\text{and}\;f_{m}=\frac{2m+1}{2\tau }$$

In the frequency domain the basis terms are the well-known sampling functions with center frequencies given in Equation (5) and null bandwidth of 2·τ^−1^. The overall frequency function is defined given the choice of the even or odd time functions in Equations (3) or (4), respectively, the basis frequency of interest, the weight of the basis function, and either the bandwidth or the time length. The coefficients, *a_n_* and *b_m_*, can take on any normalized value between −1 and 1, which determines the frequency domain spectrum. Taking on values of 1 or −1 provides a continuous spectrum and best utilization of the overall system bandwidth. This basic mathematical relationship can be used to develop a SAW RFID sensor system by using a series of properly designed Bragg reflectors, as will be discussed.

**Figure 1.**
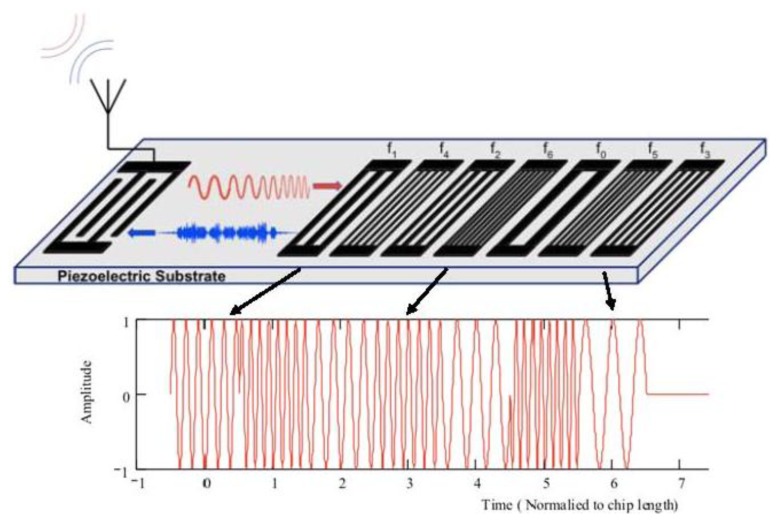
Schematic of a 7 chip SAW OFC RFID tag that can be used as the platform for a sensor. The figure depicts a chirp input time signal and the returned coded signal that is the convolution of the OFC code and chirp input signal. The lower plot depicts the ideal OFC encoded time domain in the Bragg reflectors.

**Figure 2.**
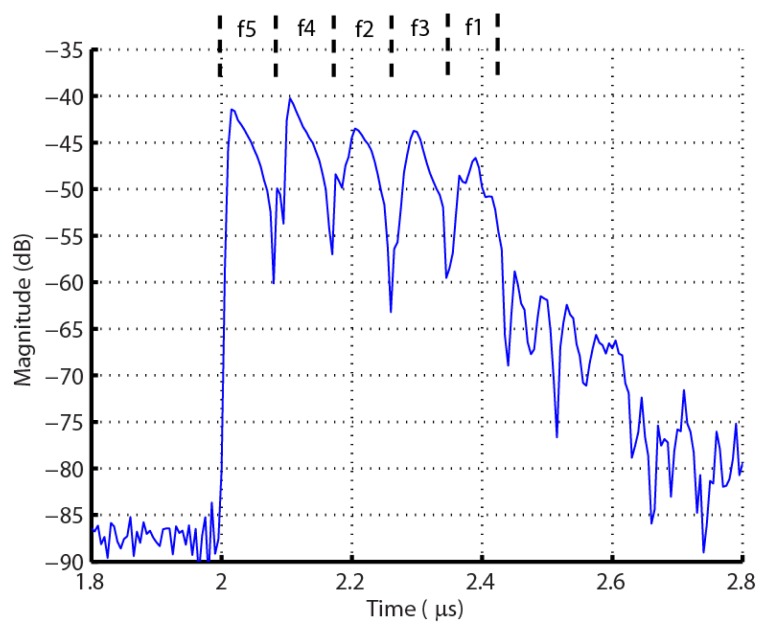
Measured |S_11_| OFC SAW tag time response, in dB, with a 5-chip shorted Bragg reflector grating. The acoustic delay allows the interrogation signal and EM reflections to dissipate before reception at the receiver.

The basic embodiment for the OFC RFID SAW tag and sensor is schematically shown in Figure 1. A wideband transducer launches a SAW based on the interrogation signal, which is convolved with the OFC coded reflector array, and is re-radiated, via the transducer antenna, back to the receiver antenna. Figure 2 shows a measured |S_11_| OFC device time domain response, illustrating the signal coded reflectivity consisting of the transducer, delay, and OFC chip encoding.

**Figure 3.**
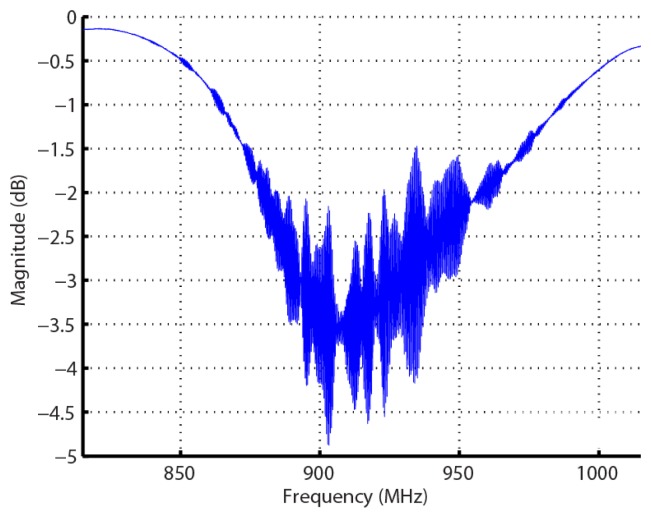
Example of a measured |S_11_| frequency OFC device response, in dB, corresponding to Figure 2. This device is centered at 915 MHz and has an approximately 92 MHz bandwidth.

The orthogonality condition, previously presented, describes a relationship between the local chip frequencies and bandwidths, embodied in each SAW Bragg reflector. The reflector-chip frequency responses are a series of nearly ideal sampling functions with null bandwidths equal to 2·τ^−1^. Each chip contains an integer number of carrier half cycles and the chip-Bragg center frequencies are separated by multiples of τ^−1^. A key enabling device feature is the fact that the nulls of adjacent Bragg reflectors align with all the peaks of the individual Bragg reflectors, which makes the SAW signal semi-transparent to all Bragg reflectors at their distinctive carrier frequency. Coding is accomplished by shuffling the chips in time, which allows both frequency and time diversity. The OFC approach produces a wide or ultra-wide band spread spectrum device, an example shown in Figure 3. The sensor information is encoded in the reflectors, time delay regions, or both. Dual tracks (in-line or parallel) can be used for enhanced coding or for multiple sensor operations.

## 3. Overall System Design Considerations

The basic system concept is composed of multiple SAW RFID-sensors (RFIDS) that may have various embodiments [9]. An antenna is connected to the sensor in an acceptable form for the application. The interrogator/receiver, often called the reader for RFID systems, sends out an interrogation signal that is received by all the SAW sensors in range. The interrogation signal, received at the sensor antenna, launches a SAW that is encoded with the RFID and is appropriately modified to also encode the sensor information, and is then rebroadcast back to the receiver. The signal is demodulated and post processed to extract the RFID and the associated sensor information.

**Figure 4.**
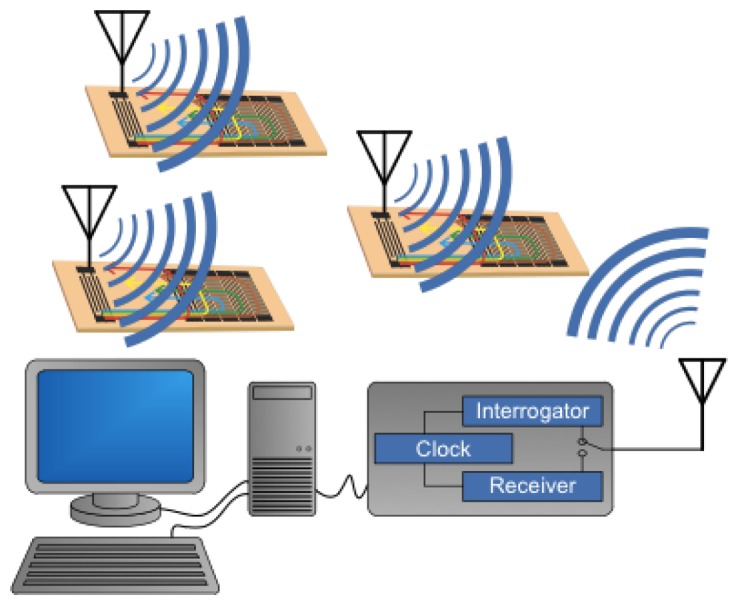
Schematic of a SAW wireless sensor system that will interrogate multiple sensors simultaneously. Receiving and identifying the RFID, the sensor information can be obtained via post processing of the received signal.

A conceptual diagram of the interrogation/receiver process is shown in Figure 4, which uses a broadband interrogation signal and a correlator receiver. A chirp (or equivalent) signal provides increased signal power over a single pulse and allows ultra wide band operation, if desired. The implementation of the actual reader hardware is more complex, but the operational principles remain the same. The near-baseband signal is post-processed through an analog-to-digital (ADC) converter and software.

There are a number of important parameters that must be considered for an optimum system design and the application and environment can often dictate the parameter choices. The following discussion will assume that the SAW antenna target must be small, and the system should have as long a sensor range as possible. No consideration will be given to government-regulated center frequency, bandwidth or output power, although these may also be constraints that need attention based on location of system. The approach is to determine system parameters for optimized overall system performance, assuming some common constraints.

## 4. Frequency and Bandwidth

To make the sensor target (SAW plus antenna) small, it is desirable to work at relatively high frequencies since this reduces antenna size while providing acceptable bandwidth and gain. As operational frequencies increase, the SAW size typically decreases, the absolute operational bandwidth increases (for a given fractional bandwidth), the acoustic propagation losses increase, and the manufactured device photolithographic resolution requirements increase. The device manufacturing constraints currently limit commercial SAW devices to less than approximately 3 GHz.

There are three key competing parameters in choice of the system operational frequency and bandwidth: the EM path loss, the SAW propagation loss and the antenna size. The EM path loss, assuming isotropic radiation, increases at 40 dB per decade change *versus* range or frequency. This parameter favors lower frequency operation. As frequency increases, the SAW substrate material losses increase; this tends to favor lower frequency operation. Each substrate is different but the trends are very similar. Devices and system presented herein will be on YZ LiNbO_3_ and will be used for illustration. The frequency dependent propagation loss constant for YZ LiNbO_3_, is given as

(6) $$\alpha \left(\text{f}\right)=0.19\text{f}+0.88{\text{f}}^{2}\;dB/\mu s,\text{with f in GHz}\;\left[10\right]$$

**Figure 5.**
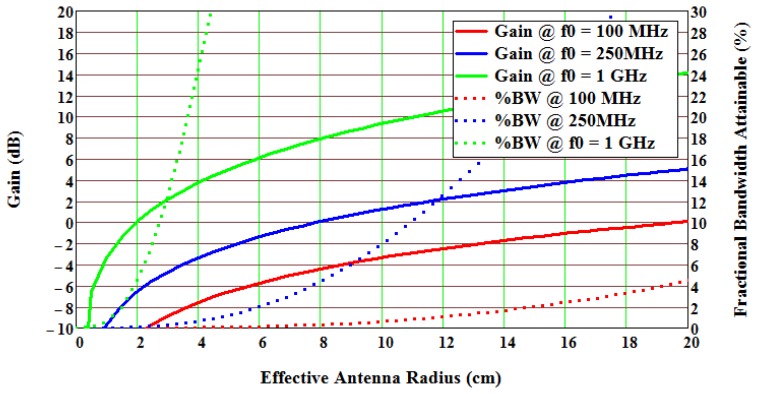
Plots of the approximate gain and fractional bandwidth *versus* effective antenna radius for an electrically small antenna, from the analysis of Wheeler [11,12]. Predicted gain Equation (solid lines) and fractional bandwidth (dotted lines) are plotted *versus* antenna radius in cm.

The loss increases rapidly above 1 GHz, and it would be desirable to operate where the loss is not a dominant factor. Also, this loss term is optimistic, since thin films and other effects often increase expected device and material loss even greater with frequency. Finally, antenna gain and achievable fractional bandwidths increase for a given antenna volume for electrically small antennas (ESA) as frequency increases; this favors high frequency operation. The antenna gain and bandwidth can be estimated for an ESA given in Figure 5, and shows that higher frequencies provide better performance with respect to both gain and bandwidth [11,12].

**Figure 6.**
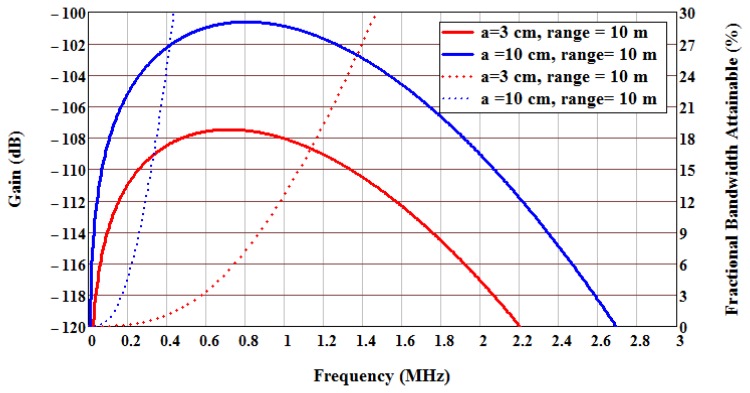
Gain and fractional bandwidth *versus* frequency considering EM path loss, SAW propagation loss, and antenna size. Two antenna sizes, 3 and 10 cm radius, are shown for illustration. The range was chosen at 10 meters, and EM propagation loss increases at 40 dB/decade with increase in range, assuming isotropic radiation. Gain Equation (solid lines) and fractional bandwidth (dotted lines) are plotted *versus* frequency.

At 1 GHz, gain greater than 0dB and a bandwidth of 13% can be achieved for an antenna radius of about inch. Higher frequencies can further increase antenna gain and bandwidth, but the SAW device propagation loss counters the advantage, and the overall target performance will be optimized in the 850 MHz to 1.5 GHz range, depending on other implementation parameter factors. Combining the parameters allows a plot of expected gain *versus* frequency and bandwidth as a function of range and antenna size, as shown in Figure 6. As observed, the optimum system operational frequency is about 800 MHz, but is relatively flat from approximately 400 to 1,200 MHz. The fractional bandwidth is much more sensitive to antenna size and frequency. For a 3 cm radius at 1 GHz, the maximum fractional bandwidth is approximately 12%, while a 6 cm radius antenna has greater than a 30% fractional bandwidth. The precise numbers are highly dependent on many parameters, but the trend and predictions are useful for design and synthesis decisions.

Based on the previous arguments, the current OFC SAW system has an operational frequency of 915 MHz and bandwidth of 74 MHz, or 8% fractional bandwidth. It was chosen to balance the conflicting parameters of SAW device's and antenna's small size, low loss, wide bandwidth, and fabrication process control. The devices at 915 MHz have a λ/4 line width of approximately 0.8 um on YZ-LiNbO_3_. High velocity materials relax manufacturing process requirements, but constraints on fabrication and propagation loss have currently limited SAW operational frequencies to below 3 GHz. High coupling materials can provide low loss operation over wide bandwidths, but typically have large temperature coefficients of frequency. The SAW OFC devices developed thus far have used YZ LiNbO_3_ since the material provides high coupling, broad bandwidths and minimal diffraction. Also, successful devices have been designed and tested on 128°YX-LiNbO_3_ and LGS materials, but will not be presented here. Several different device-antenna designs were developed. The most success was obtained with a simple folded dipole antenna fabricated on a printed circuit board which had about 0 dB gain.

## 5. Transceiver

There are several different transceiver architectures that have been previously discussed. The three most common are frequency modulated continuous wave (FMCW), pulsed narrowband, and pulsed wideband. The received signal can be processed using phase detection (narrowband), fast Fourier transform (FFT) processing, or a correlator receiver. A correlator (matched filter) system allows universal detection by software changes, for in-band frequency signals. The sensor system to be discussed here is based on a correlator receiver, software radio architecture. The output of the reader interfaces with post-processing software for extraction of sensor information. The reader pings all sensors with an RF burst and then receives the nearly concurrent SAW multi-sensor retransmitted signals. The signal is mixed down to near, or at, baseband and then sampled with an ADC. A post processor provides the correlation operation and all post processing functions. Temperature is extracted using an adaptive filter approach.

**Figure 7.**
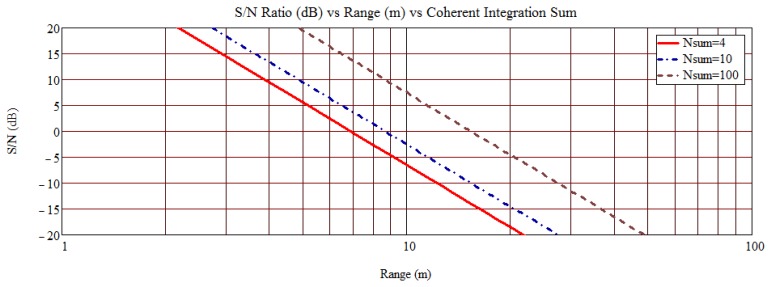
Predicted signal-to-noise (S/N) ratio as a function of range and number of synchronous integrations (Nsum) for a 915 MHz OFC SAW system. The acceptable S/N for temperature extraction is between 3 and 10 dB. Predictions yield a range of approximately 5 meters, which is consistent with current open range measurements.

The SAW OFC 5-chip sensors were designed to operate at 915 MHz with a maximum device bandwidth of 92 MHz. A synchronous transceiver (Tx/Rx), developed for NASA under an STTR contract, has a 74 MHz bandwidth, which reduced the achievable device processing gain from 25 to 15, but provides wide temperature operation. The Tx signal peak power output is approximately 28 dBm and is a stepped chirp of 700 ns duration; the Tx pulse energy is approximately 1 micro-joule. The Rx is a heterodyne design with an ADC output having a 5 μs acquisition window to receive all sensor device information. The system operates in a TDM mode with a 1 μs delay between Tx trigger and Rx trigger, which allows direct and spurious delayed Tx EM signals to dissipate. The SAW devices are designed with a 1 μs acoustic delay to match the transceiver TDM operation. The open range signal decreases at 40 dB per decade for a fixed equivalent isotropically radiated power (EIRP). Based on current device and system configuration, the received signal-to-noise (S/N) ratio is estimated, shown in Figure 7, as a function of range and synchronous interrogations. Assuming a 5–10 dB S/N is required for sensor parameter extraction, then a range of approximately 5–15 meters for integrations from 4 to 100 is expected; consistent with current temperature extraction data. The data transfer from the analog-to-digital-converter (ADC) transfer buffer currently limits acquisition times to nearly 0.5 s, but the acquisition time can be greatly increased with the use of a faster data bus, programmed field programmable gate array (FPGA) or onboard processor for post processing prior to data transfer. The ADC can sample at the IF frequency or subsampled, consistent with the Nyquist rate for the signal bandwidth. Subsampling reduces the ADC sampling rate, but the sample bandwidth still needs to be fast; consistent with the signal carrier frequency to obtain accurate sampled time amplitudes.

The combination of OFC device and custom post processing software provide fast and accurate RFID and temperature extraction. The software processing of a single sensor is currently <10 ms and faster post processing software development is continuing. It is anticipated that software code will ultimately allow all sensors to be acquired and temperature extracted in <1 ms. The combination of hardware and software enhancements should allow kHz acquisition rates in future systems. Finally, analysis for higher performance systems indicate that 100s of meters should be possible with high gain antennas, greater output power, greater precision ADC's and lower noise figure receivers.

## 6. SAW OFC Signal Processing

An important feature of the SAW technology is the ability to perform fairly complex signal processing. The devices can operate over wide ranges of RF frequencies, have variable bandwidths, and can produce complex time waveforms. By properly tailoring frequency, time, bandwidth, phase and delay, a communication link is established, an RFID is encoded, and sensor information is embedded. If using just a SAW die and antenna, all this is accomplished passively and wirelessly. There are three most common types of SAW RFID sensors approaches: a SAW resonator, a uni-carrier frequency code division multiple access (CDMA) or pulse position reflector, and the OFC multi-carrier CDMA or pulse position reflector. The later two devices are often referred in the literature as delay line sensors. There are some perturbations on these approaches, but they can generally be classified under one of the three.

A SAW resonator encodes only in frequency, achieving its coding diversity in only one domain. The system allowed bandwidth is divided into sub-bands, and each device is orthogonal so long as it stays within its sub-band. Usually there is little inherent device delay, so multiple sensors would all overlap in the time domain and can only be RFID, or sorted, in the frequency domain. The device can have high Q, which translates to narrow bandwidth and low loss SAW devices, which are attributes for antenna design and increased range. The devices can suffer from fading effects and limited coding diversity.

The uni-carrier frequency CDMA approach uses pseudo-random code sequencing to achieve encoding, similar to any conventional communication link of its kind. This technique uses multiple chips that can have an arbitrary time delay or frequency phase relation, based on the position of multiple chip reflectors. The signal bandwidth is determined by the lesser of the transmission signal, receiver, or sensor bandwidth. The detectable RFID is established by the degree of orthogonality of the signal produced by each sensor compared to all others; often determined by the cross-correlation properties of the codes. As an example, a very large code ensemble has been developed for RFID purposes using a 32-bit pulse position modulation-coding scheme [13]. This uni-carrier frequency approach at 2.4 GHz has insertion losses of 30–40 dB due to the SAW device implementation constraints within the Bragg reflector scheme.

The SAW OFC multi-carrier approach, as previously described, use a combination of time delay and pulse position diversity, and Bragg reflector frequency diversity. Various embodiments allow low loss, approximately 10 dB has been demonstrated, and orthogonality can be optimized in both time and frequency, as will; be discussed in the next section.

## 7. OFC Coding

Given the accessibility of both time and frequency diversity for OFC, a number of different approaches and embodiments have been explored and offer various advantages and disadvantages. A brief review of an approach using block coding (this is where all the chips are contiguous in time) will be presented, which provides many of the key coding element considerations. The analysis presented is theoretical and ideal; the SAW embodiment will determine the applicability of the theory to actual device results. The approach will have both frequency and time diversity that provides a systematic way of implementing a code in a SAW device embodiment.

Given a time function *g_bit_(t)*, having a time length *τ_B_* defined as the bit length, the bit will be divided into an integer number of chips such that:

(7) $$\tau_{B}=J\;\cdot \;\tau_{c}\;\text{where J}=\#of\;\text{chips}$$

and the chip interval, *τ*_c_, is the time interval for the basis set. Given a definition of each chip as *h_cj_(t)*, a bit is defined as the sum of J chips as:

(8) $$g_{\text{bit}}\left(t\right)=\sum_{j=1}^{J}w_{j}h_{cj}\left(t-j\;\cdot \;\tau_{c}\right)$$

where each chip, *h_cj_(t* − *j*·*τ_c_)* is contiguous without time overlap and the bit weight is *w_j_*, In general, multiple local carrier frequencies are possible in each chip depending on their weighting coefficient. In general, each chip can be defined as:

(9) $$h_{cj}\left(t-j\;\cdot \;\tau_{c}\right)=\sum_{m=0}^{M}b_{jm}\;\cdot \;\cos\;\left(\frac{\left(2m+1\right)\;\cdot \;\pi \;\cdot \;\left(t-j\;\cdot \;\tau_{c}\right)}{\tau_{c}}\right)\;\cdot \;\text{rect}\;\left(\frac{t-j\;\cdot \;\tau_{c}}{\tau_{c}}\right)$$

To generate the required signal, let *b_jm_* = *0* for all m, except *m* = *C_j_* Where *1* ≤ *C_j_* ≤ *M*. Then:

(10) $$h_{cj}\left(t-j\;\cdot \;\tau_{c}\right)=b_{j}\;\cdot \;\cos\;\left(\frac{\left(2C_{j}+1\right)\;\cdot \;\pi \;\cdot \;\left(t-j\;\cdot \;\tau_{c}\right)}{\tau_{c}}\right)\;\cdot \;\text{rect}\;\left(\frac{t-j\;\cdot \;\tau_{c}}{\tau_{c}}\right)$$

where each chip has a single local carrier frequency *f_cj_* = *(2C_j_* + *1)/2*·*τ_c_* and *b_j_* is the chip weight. In order to build the desired time function, the following design rules are used: (1) *b_j_* = ±1 for all *j*, (2) the bit null bandwidth is $BW_{\text{bit}}=J\cdot 2\cdot \tau_{c}^{-1}$, and (3) *C_j_* is a sequence of unique integers, where *f_cj_* form a contiguous, non-repetitive set. The local frequency of adjacent chips that are contiguous in frequency need not be contiguous in time, in fact, the time function of a bit provides a level of frequency coding by allowing a shuffling of the chip frequencies in time, as depicted in Figure 1, where *f_cm_* ≠ *f_cn_* for all m ≠ n, and there are an integer number of half wavelengths in each chip. The local chip frequencies are contiguous in frequency but are not ordered sequentially in time.

The given chip sequence represents the orthogonal frequency code for the bit. If there are *J* chips with *J* different frequencies in a bit, then there are *J*! possible permutations of the frequencies within the bit. A signal can be composed of multiple bits, with each bit having the same OFC or differing OFC. For the case of a signal, *J* chips long, *b_j_* = 1, and having a single carrier frequency, the signal is a simple gated RF burst τ_R_ long.

In addition to the OFC coding, each chip can be weighted as ±1, giving a pseudo noise (PN) code in addition to the OFC, namely PN-OFC. This does not provide any additional processing gain since there is no increase in the time-bandwidth product, but does provide additional code diversity for tagging. For conventional PN coding, the number of available codes is 2^J^. When using PN-OFC coding, the number of available codes is increased to 2^J^·*J*!; though all code combinations are not useful and the useable number is greatly reduced—similar to PN coding.

**Figure 8.**
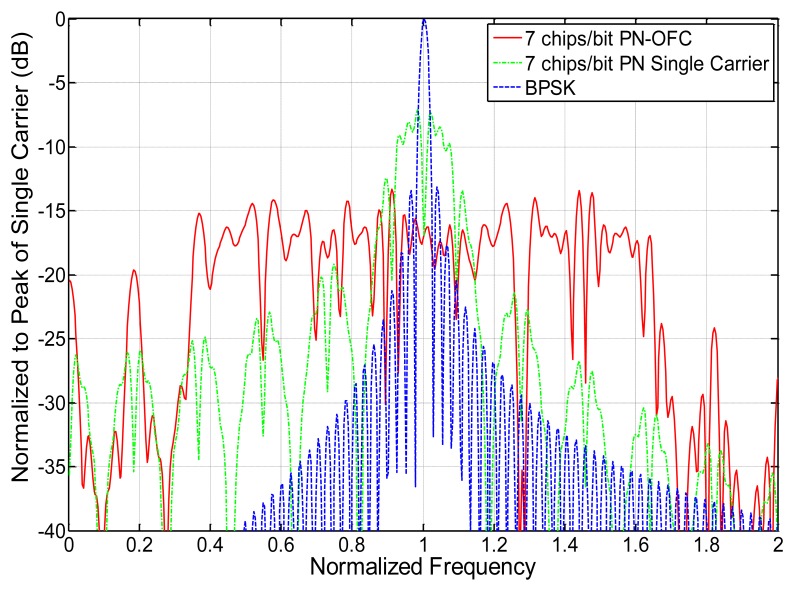
Frequency responses of seven chip OFC, seven chip Barker code, and single frequency carrier (BPSK), each with identical time lengths.

As a brief comparison of the typical signal formats for frequency and time encoding and their correlation properties, some ideal plots were generated and are presented. Figure 8 shows the ideal bit power spectral density of a seven chip OFC, seven chip Barker code PN, and uncoded single-frequency carrier signal with time functions normalized to unity and having identical impulse response lengths. The uuencoded single carrier is narrowband and has greater peak amplitude at center frequency than the PN (−9 dB) and OFC (−17 dB) signals, but all signals have the same total energy. The bandwidths of the PN and OFC signals are 7 and 49 times greater than the single frequency carrier bandwidth, respectively, as expected due to the spread spectrum nature of the signals. The power spectral density is lowest for the OFC signal due to its wide bandwidth.

**Figure 9.**
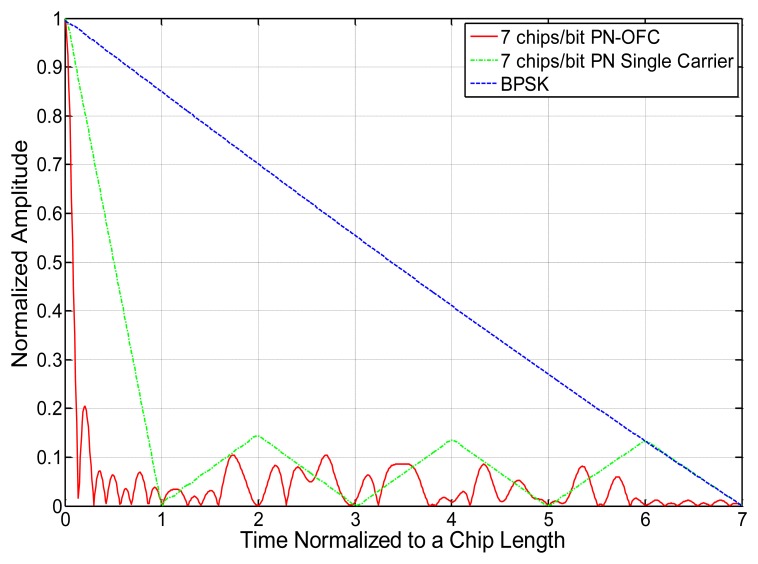
Time autocorrelations of a seven chip PN-OFC, seven chip PN (Barker code), and single-frequency-chip (BPSK) signal having identical time lengths. Only half of the autocorrelation is shown due to symmetry. The peak correlation amplitude is the same, but the main compressed pulse width is inversely proportional to bandwidth.

Figure 9 shows the autocorrelation time functions of the Figure 8 signals. The peak autocorrelation is exactly the same, given the identical time amplitude and signal lengths, but the compressed pulse widths for the coded signals are narrower than that of the uuencoded single carrier, as expected due to their wider bandwidths. This provides the measure of processing gain Equation (PG), which is the ratio of compressed pulse width to bit length, for equal energy signals. The signal bandwidth determines the main compressed pulse width, and the encoding determines the auto- and cross- correlation time sidelobes.

An important issue to be addressed is minimization of code collisions from multiple sensors. The problem is the overlap of energy from various excited sensors being simultaneously received at the reader. Since the devices are passive, their placement and temperature variations yield random delay reception; resulting in completely asynchronous multi-sensor reception at the transceiver, which reduces most advantages of many classical coding techniques. Maximum diversity must be used in a multisensory system in order to be able to accurately detect sensor data. The principle modes of diversity are frequency, time, spatial placement (or antenna focusing), antenna polarization, and code sequencing. Frequency can be portioned between sensors, but this reduces code perturbations for RFID. In general, many RFID sensors may occupy the same frequency band and only limited frequency partitioning can be done. Time diversity is accomplished by moving a block of chips into different time bins or slots by physically modulating placement of chips on the substrate. This can be practically implemented on the SAW substrate, with the ultimate constraint being the device length. Antenna focusing and polarization are well known and apply similarly here.

**Figure 10.**
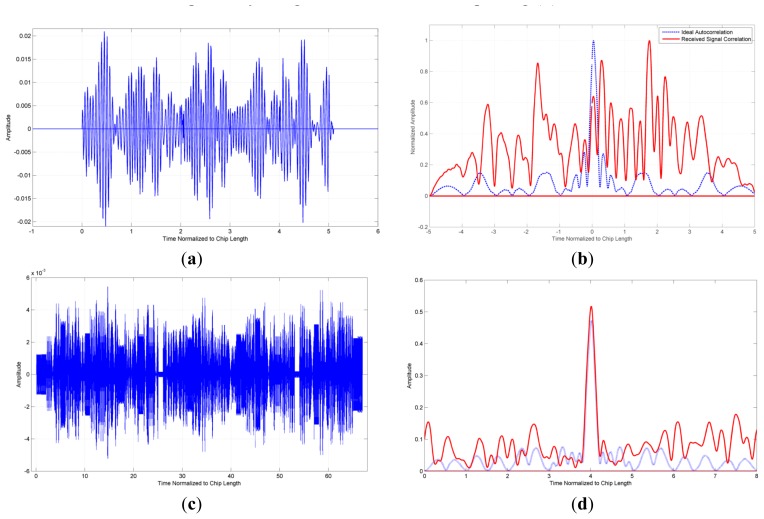
Examples of code collisions for 32 code set ideal OFC system for two cases, relative amplitude (vertical) *versus* time in units of chip length (horizontal). Upper—all 32 codes arrive simultaneously at receiver (**a**) and the resulting matched filter correlation cannot be extracted (**b**). Lower—the 32 codes are time division multiplexed, spreading the response over a larger time window (10×) but reducing energy overlap from adjacent codes (**c**). The matched filter correlation can be extracted and the cross correlation interference is acceptable by using the time division multiplexing (**d**).

For OFC, code sequencing is the availability to shuffle the chips in time, and adding some levels of binary coding. A simple example is illustrated in Figure 10, which is based on a 32-code OFC set, assuming 32 sensors, having five chips in the code. There is no time division multiplexing (TDM) between codes and all 32 codes are assumed to arrive simultaneously. The desired correlation of one code from a correlator receiver is completely masked by the multi-code self-interference. By allowing some time code overlap but staggering the codes so only a few overlap in any given chip-time-sample, which is one form of TDM, the codes are spread over a larger time window; resulting in an unambiguous correlation peak in time. The cost is that the device with the longest delay must have a longer die size and more propagation loss. This can be balanced in a system by placing long delay devices closer to the reader, if allowed to be predetermined. The code results in Figure 10, however, may be very optimistic. Multiple system filters, dispersion, sensor frequency offsets, and arbitrary time delay offsets can further decrease sensor parameter extraction due to multi-sensor intersymbol interference.

Minimization of auto- and cross- correlation sidelobes is also important to reduce interference at the desired correlation peak and to provide sufficient peak-to-sidelobe ratio from inter-chip interference and noise. Offset of the correlation peaks in time, TDM, is used to set windows of interest to lock onto the desired signal. Codes must be sufficiently diverse to account for time delay variations due to temperature and measurand effects. For the approach presented, the devices use time diversity with offset of 5 chips per code, which causes manageable time overlap between devices. System design must consider the convolution of all transfer functions that result in lengthening of the ideal OFC chip length and their effects on identifying the sensor, as well as extraction of the measurand data.

Because of the asynchronous nature of passive SAW sensors, the lack of an “active hand-shake” between the transceiver and sensor, and the fact that frequency and time variations are the parameters for encoding sensor changes, unique coding techniques have been and continue to be developed. Typical code sets used in common communication systems, unfortunately, do not work well due to the SAW sensor system asynchronous nature, the finite time domain code lengths due to realizable die lengths, and the simultaneous reception of all sensors active within an interrogator's observation window. Use of all the diversity options previously mentioned, should allow 50–100 sensors to be simultaneously received, identified and processed. The maximum SAW die size is a function of the number of sensors used, the coding approach, and the system bandwidth. The ideal OFC coding format determines the chip length *versus* chip bandwidth, where:

(11) $$BW_{\text{chip}}=\tau_{c}^{-1}.$$

If the chips on any one sensor are contiguous in time, *i.e.*, the chips adjoining each other in time, then the code length is given as:

(12) $$\tau_{\text{sensor}}=N_{\text{chip}}\cdot \tau_{c}$$

**Figure 11.**
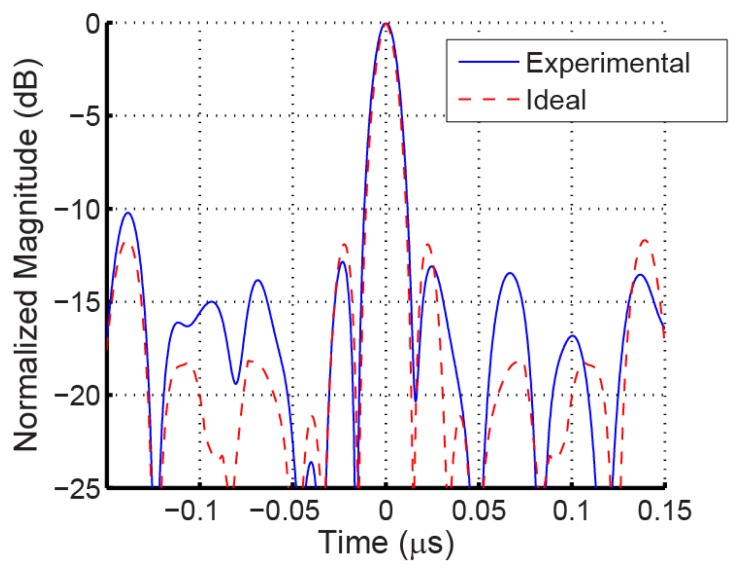
Comparison of ideal and RF probed measured time domain matched filter response, in dB, for a 5-chip 915 MHz OFC SAW device. The SAW device's non-ideal time response, when correlated with the ideal matched filter, provides good correlation sidelobes.

For the current system, the chip bandwidths are approximately 18 MHz, for a code bandwidth of approximately 92 MHz, and a pulse length of approximately 56 ns. Each sensor has a total code length of five chips, or 278 ns. minimum SAW transit delay between transducer and the closest device OFC grating is 1 μs or 3.5 mm, which is set based on the interrogation signal length and used to permit multi-EM reflections to subside. An example of a 5-chip time correlation, measured from a SAW OFC device and predicted from ideal signal theory, using the ideal signal as the reference, is shown in Figure 11. The OFC has much better sidelobe rejection than CDMA for five chips due to the wider band spread spectrum effect of the multi-carrier chips. In a 5 μs window using conventional OFC sensors without spatial or antenna diversity, the number of sensors may be limited to 10–25 units; cross-polarized antennas will double that number. Alternative coding techniques need further research to provide a workable set of 50 to 100 sensors. Although there are some publications on coding techniques for passive wireless sensors, most do not address the code collision effects with environmental changes that cause delay and fading with resulting loss of synchronization.

## 8. Coherent Correlator and Matched Filter Approach

For purposes of this discussion, it is assumed the hardware is capable of providing the following:

- The transmitter and receiver are used in a time duplexed mode, opposing on-off state.
- The transmitter and receiver are operated in a synchronous mode for switching and integration.
- The interrogation signal is a wideband, time-pulse.
- The transceiver outputs a windowed time domain Equation (or frequency domain sweep) to a post-processor.

Post processing of a temperature sensor involves extracting the sensor's RFID as well as the temperature information, and these operations are accomplished concurrently. A change of device temperature varies the SAW velocity due to the material's temperature coefficient of delay (TCD) and translates into scaling of the SAW device frequency and the time domain responses. To extract the change in the SAW surface velocity, a set of matched fi chan *versus* code and temperature are generated, which is essentially the same function but scaled in time and frequency using Fourier Transform properties, given as:

(13) $$\begin{array}{c} {\text{h}}_{\text{correlation}}\left(\text{t}\right)={\text{h}}_{MF}\left(\alpha \;\cdot \;t\right)⊛h_{\text{OFC SAW}}\left(t\right)\\ \text{where}\;h_{\text{correlation}}\left(t\right)=\text{time correlation signal},\\ h_{MF}\left(\alpha \;\cdot \;t\right)=\text{the matched filter response for the coded device at a given temperature},\\ ⊛=\text{convolution operator},\\ h_{\text{OFC SAW}}\left(t\right)=\text{SAW sensor received time domain response},\\ \text{and}\alpha \text{is the frequency scaling factor}. \end{array}$$

**Figure 12.**
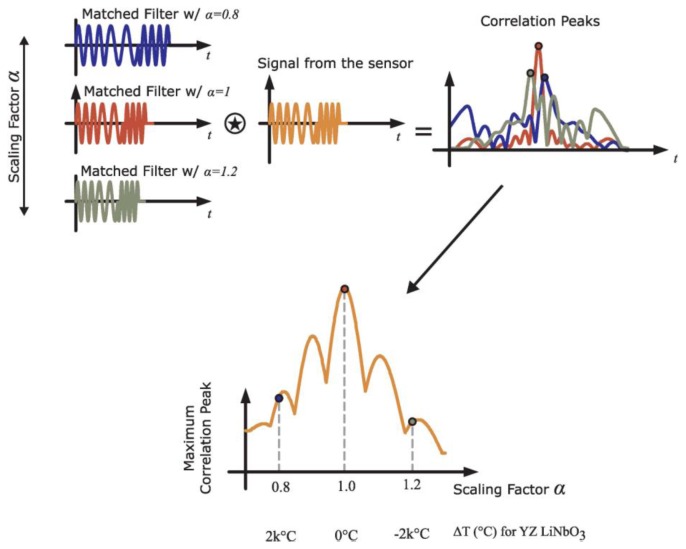
Principle of operation of the adaptive matched filter approach to maximize the correlation waveform and extract the SAW sensor temperature. The frequency scaling factor changes and matched filters are generated, examples shown for **α** = 0.8, 1, and 1.2. The convolution of the matched filter with the sensor signal produces a correlation response. The peak of the correlation response is plotted *versus***α** and the maximum value is mapped back into temperature for a given substrate.

The frequency scaling factor, α, is swept over the required expected temperature range such that h_correlation_(t) is maximized, which corresponds to the temperature of the sensor [5,7]. At the ideal designed sensor temperature, α = 1, and it deviates linearly *versus* temperature at -94 ppm/°C for YZ LiNbO_3_. Figure 12 shows a sketch of the mathematical process of the temperature extraction algorithm. The device code and the adaptive matched filter can be used for further processing to obtain RFID and sensor data. The time delay of the received signal can then be obtained be further post processing.

In general, a signal or waveform may not have a well-defined peak or even have constant group delay. Encoding may be near-ideal, but inevitably there is distortion due to system and noise sources. For purposes of this discussion, the ideal matched filter (MF) is the time-reversed replica of the received signal being analyzed. The matched filter is a convolution process, while the system correlation process is the product integration of the ideal transmitted signal with the received signal. The mathematical operations are different, but the two terms are often used synonymously, and post processing provides similar information [14]. The MF has a number of useful properties:

- The MF provides the highest signal to random-noise ratio.
- The ideal MF waveform is always a symmetric time domain pulse compression, regardless of the nature of the signal. In the frequency domain, the signal response is non-dispersive.
- The peak of the time domain compressed pulse always occurs at the center of the MF time response. If detecting in the time domain, the pulse is well defined and easily detectable.
- The MF is always non-dispersive, even for amplitude, phase or frequency modulated signals. The MF yields a linear phase, band-limited frequency response.
- If the signal phase delay is functionally included as part of the received signal, then the MF is purely real in both domains, with the peak compressed time pulse at t = 0 s, and the signal having no delay.

At post-processing, the quadrature noise may be eliminated, increasing the effective S/N by 3 dB yielding an optimized detection condition.

**Figure 13.**
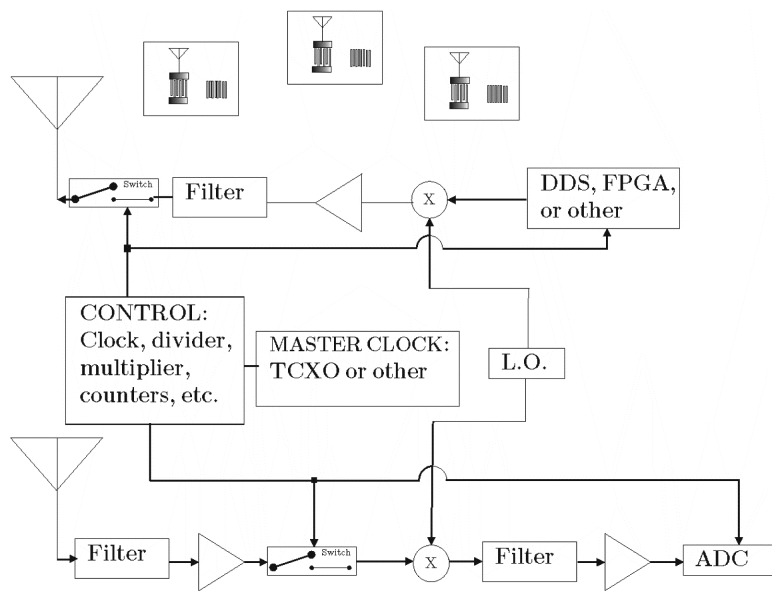
A heterodyne coherent correlator transceiver block diagram for use in a multi-sensor SAW system with 3 SAW sensors within the antenna range. The system assumes a wide-band pulsed transmit signal, and time duplexed between transmit and receive cycles. The output from the ADC is input to a post processor that is typically a software based signal-processor.

Figure 13 shows a simple block diagram of a heterodyne synchronous transceiver system. This is but one type of receiver architecture that provides time domain data for post-processing, and other transceiver designs are possible. The analog-to-digital converter (ADC) output signal is time windowed and frequency band limited to obtain the multiple sensors data that are in the receiver's view. The ADC output is transmitted to a post-processor or computer for parameter extraction.

A typical requirement for demodulation of a SAW RFID, RFID sensor, or single-sensor device is to detect either the unique frequency (resonance-based), the relative delay of multiple time domain pulses (CDMA reflectors), or multiple orthogonal frequency modulated time domain pulses (OFC reflectors). The MF correlator approach is applicable for any modulation format and many system types. It is assumed that either the frequency or time domain data are provided over a sample window from the receiver hardware, such as an ADC. A post processor will provide FFT and all other digital signal processing capabilities.

Many previous approaches have extracted time delay using the signal time domain impulse response, which is intuitively the most obvious and direct. Extracting the signal group delay using the frequency phase response is another common approach. Multiple pulses and amplitude, frequency and phase dispersive pulses become difficult to determine time delay information on the ensemble signal. If the time response is given from an ADC, time windowing may be appropriate to remove spurious signals from the system or environment, and then the data is transformed to frequency for the correlator time delay extraction process. The signal at the receiver, H_R_(f), is assumed to have the form:

(14) $$H_{R}\left(f\right)=\sum_{i=1}^{N}\left[H_{i}\left(f\right)+E_{i}\left(f\right)\right]\;\cdot \;e^{-j2\pi f\tau_{Di}}+H_{\text{SN}}\left(f\right)+H_{CN}\left(f\right)$$

The ideal-model coded signal from each device, *H_i_(f)*, is that expected for the matched filter correlation. The error signal, *E_i_(f)*, is associated with each device due to device implementation and system effects. The error produces amplitude, phase and delay distortions with respect to the ideal signal. If fading is ignored and the channel is assumed stationary, *E_i_(f)* is stationary. The assumed random stationary noise, *H_SN_(f)*, includes AWGN, quantization and other sources, and *H_CN_(f)*, which includes all constant additional noise and interference sources. These can be thought of as external jammers, or signals produced within the transceiver, which are constant with time. The i^th^ sensor's actual delay, τD_i_, is that measured accurately with a VNA or other source.

It is understood that demodulation of each signal can be done in any order; but is assumed to be accomplished sequentially. Assume a matched filter process, such that:

(15) $$G_{i}\left(f\right)=H_{R}\left(f\right)\;\cdot \;H_{i}^{*}\left(f\right)$$

The explicit frequency dependence will be dropped from the notation for most terms for simplicity, unless needed. For illustration of the technique, sensor #1 will be used as the device for detection. Extraction of delay information using a matched filter approach for sensor #1 yields:

(16) $$G_{1}=H_{1}\;\cdot \;H_{1}^{*}e^{-j\omega \tau_{D_{1}}}+E_{1}\;\cdot \;H_{1}^{*}e^{-j\omega \tau_{D_{1}}}+\sum_{i=2}^{N}\left(H_{i}+E_{i}\right)\;\cdot \;H_{1}^{*}e^{-j\omega \tau_{D_{i}}}+H_{CN}\;\cdot \;H_{1}^{*}+H_{SN}\;\cdot \;H_{1}^{*}$$

The first term represents the desired frequency domain MF (auto-correlation) of the received signal with its ideal reference. The error term $E_{1}\;\cdot \;H_{1}^{*}e^{-j\omega \tau_{D_{1}}}$ is due to system, channel or device non-ideal distortion effects of the sensor being demodulated. The summation term $\sum_{i=2}^{N}(Hi+Ei)\;\cdot \;H_{1}^{*}e^{-j\omega \tau_{D_{i}}}$ represents all other sensor received signals at the antenna. These could represent a large in-band, noise-like term, depending on the number of sensors and the inter- and intra- sensor code collisions. The remaining terms $H_{CN}\;\cdot \;H_{1}^{*}+H_{SN}\;\cdot \;H_{1}^{*}$ represent the effects of random thermal noise and any jammers. All of the error and noise terms will result in determining the minimum detectable signal within the correlator receiver. If an FFT is taken, the time domain MF (auto-correlation) peak is obtained, along with the other noise terms that may distort the desired peak response. Suitable algorithms can be applied to the data to extract the time delay. If peak time domain detection is used, then the accuracy of the extracted delay often relies on single point determination or fitting algorithms to pulse shape. Zero padding may be used for interpolation to increase peak extraction accuracy.

Initially, each device's approximate time delay needs to be extracted. An estimate of the delay time using the device design parameters, passband frequency phase-slope, or in the time domain by using the approximate correlation peak, is obtained. Multiplying both sides of the equation by the estimated delay, *τ_D1_* + *Δτ_E_*, yields:

(17) $$\begin{array}{c} GT_{1}=G_{1}e^{+j\omega \left(\tau_{D_{1}}+\Delta \tau_{E}\right)}={\left|H_{1}\right|}^{2}e^{+j\omega \Delta \tau_{E}}+E_{1}\;\cdot \;H_{1}^{*}\left(e^{+j\omega \Delta \tau_{E}}\right)\\ +\sum_{i=2}^{N}(H_{i}+E_{i})\;\cdot \;H_{1}^{*}e^{-j\omega (\tau_{D_{i}}-\tau_{D_{1}}-\Delta_{\tau_{E}})}\\ +H_{1}^{*}e^{+j\omega (\tau_{D_{1}}+\Delta_{\tau_{E}})}+H_{SN}\;\cdot \;H_{1}^{*}e^{+j\omega (\tau_{D_{1}}+\Delta_{\tau_{E}})} \end{array}$$

where Δτ_E_ represents the error in the estimated delay *versus* the actual device delay. The functional description in Equation (4) now can be manipulated to extract the actual delay.

The desired matched filter signal response is contained within the first terms of GT_1_. Define the matched filter response for the desired sensor as:

(18) $$MF_{1}={\left|H_{1}\right|}^{2}\left(e^{j\omega \Delta \tau_{E}}\right)={\left|H_{1}\right|}^{2}\;\cdot \;\left[\cos\left(\omega \Delta \tau_{E}\right)+j\;\cdot \;\sin\left(\omega \Delta \tau_{E}\right)\right]$$

As Δτ*_E_* → 0, *MF*_1_ → |H_1_|^2^ which indicates that the estimated delay, τ*_D_*_1_, is exact; then *MF*_1_ is purely real, independent of frequency, and maximum valued. Using the adaptive matched filter concepts allow the extraction of both frequency variations with temperature and the extraction of device time delay.

## 9. Experimental Results

### a. SAW Device Design Summary

The SAW sensors all fabricated on YZ LiNbO_3_ using well know fabrication techniques. Devices are fabricated using a liftoff technique, aluminum electrodes of approximately 1,000 angstrom thickness, and have nominal line widths of 0.8 μm. The nominal center frequency is 915 MHz, a broad band quarter-wavelength interdigital transducer is used for SAW coupling and connected directly to the antenna. The sensors each use five chip OFC Bragg quarter-wavelength aluminum reflectors, having approximately 50 electrodes each. Each has its own unique OFC code for identification. Devices used with the printed circuit board dipole antennas are mounted in surface mount packages, ball bonded, and soldered to the antenna connections.

### b. Transceiver and Antennas

**Figure 14.**
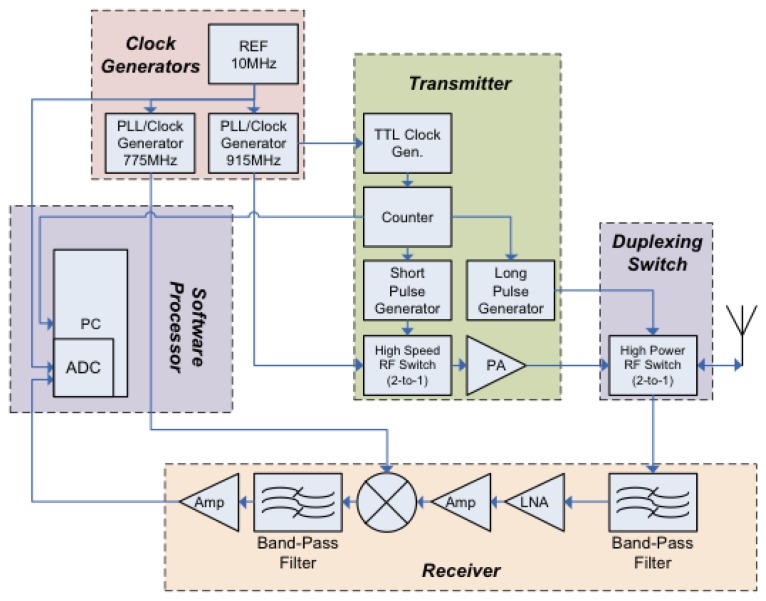
A simple block diagram of an implemented RF transceiver at 915 MHz.

There have been many previous publications on various approaches to SAW sensor transceivers, but most addressed single devices [15–19]. Several different transceivers, also referred in RFID as the reader, have been built in the course of the OFC system research. Figure 14 shows a typical simplified block diagram of a 915 MHz system used in the investigations. There is a wideband-transmitted pulse used to excite the sensors. A duplexer switch is used prior to the antenna to allow the transmitted pulse to radiate out the antenna while the receiver is “off”, and a delay of approximately 1 μs when the transmit switch is turned “off” and the receiver channel is “on”. The SAW sensors have a minimum of 1 μs acoustic delay designed within the die. The received signal is then captured by the analog-to-digital converter (ADC) and routed to the processor (laptop computer) for post processing to obtain the RFID and extract the sensor information. All of the sensor device results used a version of the synchronous correlator transceiver described. Software is adapted to the individual sensor to extract the required data.

**Figure 15.**
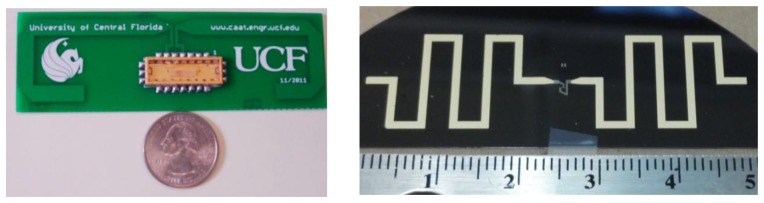
Left—Photo of a packaged 915 MHz SAW sensor and antenna [20]. The antenna is a simple folded dipole on a PCB connected to the device through the surface mount package. Right—Integrated SAW/antenna on black YZ-LiNbO_3_ using an electroplated gold film for the meander-line dipole pattern. The SAW sensor, seen in-between the dipole arms, requires line width resolution of approximately 0.8 μm, compared to the antenna having an order of magnitude larger dimensional resolution. The SAWtenna is rugged and useful for many applications requiring no mechanical bonds [21].

Two different sensor target embodiments were designed. The first uses a PC board, open sleeve folded dipole antenna and the surface mount packaged (SMP) SAW is soldered to the board, shown in Figure 15. The antenna with SMP device was 125 mm × 55 mm on a 32 mil FR4 copper clad PC board [20]. The measured antenna gain, in a 50-ohm system, is approximately 1.5 dB and had a bandwidth of 140 MHz. The second approach uses a fully integrated SAW sensor and antenna on the LiNbO_3_ substrate, as shown in Figure 15 [21]. The first prototype is less efficient than the PC board antenna, but was successfully interrogated from several meters. This approach is termed a SAWtenna, and offers great promise for future integration. The SAWtenna removes all external bonds, solder and other attachments, which increases reliability for insertion in high temperature or strain sensor applications. Both devices and antenna were designed to yield low reflection loss without external matching.

**Figure 16.**
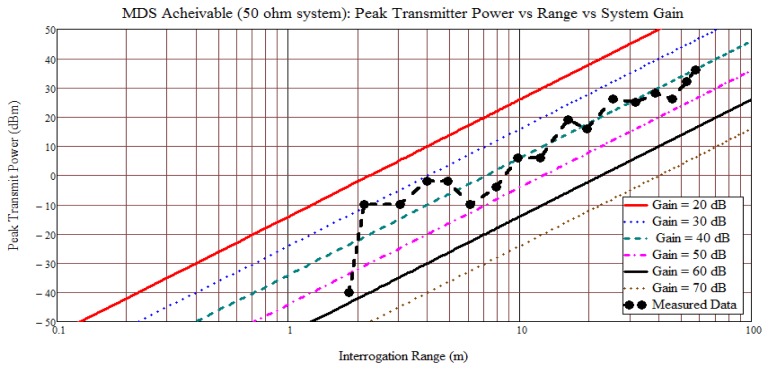
Data is measured in a hallway approximately 2.1 meters wide. Transmit signal is a single, 700 ns, 915 MHz chirp pulse. The OFC SAW device uses five chips, each with an approximate 18 MHz bandwidth. Effective SAW device processing gain is 15 in the measurement system.

Figure 16 shows an early experimental plot, data taken September 2010, of the predicted range *versus* estimated peak-Tx output power *versus* estimated system loop gain. A Yagi with 9 dBi gain was used on the Tx and Rx with 38 dBm Tx peak output power. Data was taken as an inline variable attenuator was changed and the range was based on detectable RFID above the noise; not sensor temperature extraction. The measured range for a single sensor taken in a 2 × 4 meter hallway is plotted, and fading effects are observed while the linear regression on the range data yields a slope of 38.7 dB/decade; close to the open range predictions. The greatest range achieved for this initial system measurement was approximately 60 meters with a single sensor under test and is probably strongly affected by waveguiding, but demonstrates the long-range possibilities in a given environment. The simple initial test condition attested to the possibilities of long- or short-range sensor measurements using wideband passive SAW sensors and a pulsed TDM correlator receiver in various environments.

### c. Temperature Sensors

Reindl provides a review of a wide selection of demonstrated sensors, most of which were for single device detection [22]. In 2010, initial data measurements on a 915 MHz OFC four (4) sensor system were first shown [23], with range of approximately 3–4 meters and extraction of temperature accuracy to within approximately ±5 °C The limitations at that time were principally the extraction software, which used simple peak correlation pulse detection techniques. The current data processing and associated software approaches, as described earlier, have increased processing speed, accuracy and range; yielding a 100 fold increase in processing speed, ±2° Cover a 300 °C temperature window, and range of 10–20 meters.

To determine if the OFC sensors could be used within an enclosed metal structure for applications in wing, fuselage, or other similar enclosures, tests were performed with four sensors placed within an approximately 2 cubic-foot metal toolbox (simulating a small Faraday cage); all sensors were RFID identifiable and temperature extracted within ±2 °C. This test demonstrated that the OFC SAW designs and TDM system operation aids in reducing fading effects, even in very small, EM-reflective environments.

**Figure 17.**
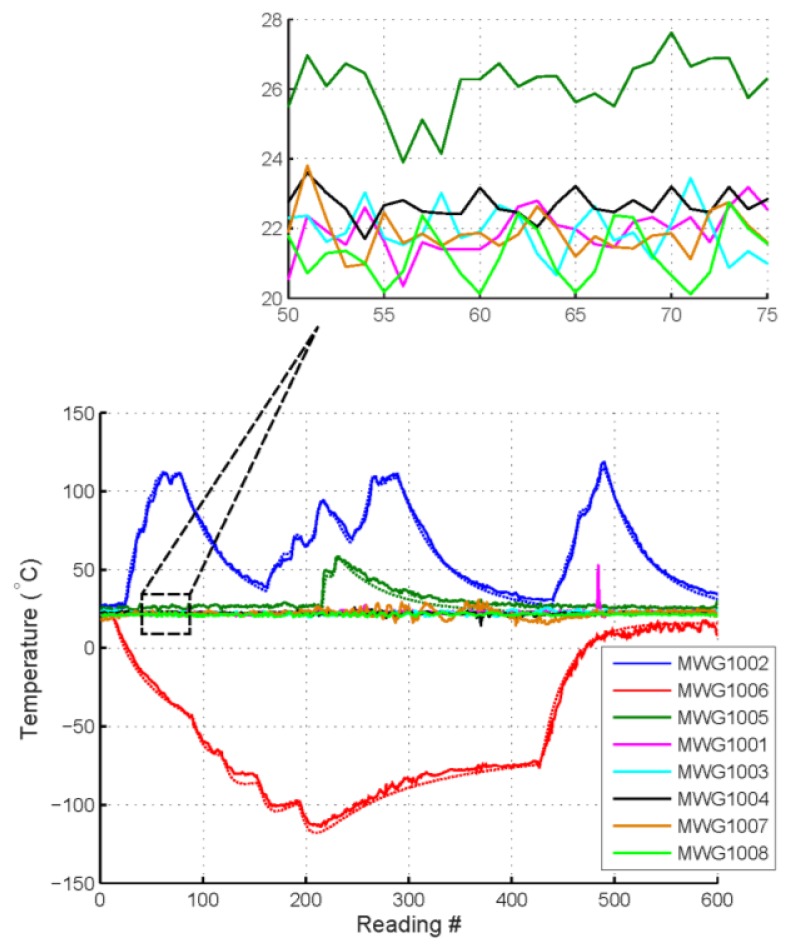
Wireless OFC 8-sensor synchronous temperature sensor system data extraction in an open-atrium from approximately 4 meters in range. Synchronous integration of four sweeps per sample per data point. Two sensors are repeatedly heated or cooled; then allowed to return toward ambient temperature. The heated and cooled sensors have an attached thermocouple to illustrate the tracking.

An open range experiment for extracted temperature data, shown in Figure 17, was taken while physically placing eight OFC sensors randomly over a range of 4 meters. There are a few spikes in the data when physical movements caused loss of acquisition, time is continuous and no data points have been removed. Sensors were heated or cooled during the real time dynamic operation and data collection. Data is measured simultaneously of all sensors with four integration acquisition sweeps. Five (5) sensors were kept at room temperature while three (3) sensors underwent temperature variations over time.

### d. Multi-Parameter Sensing

**Figure 18.**
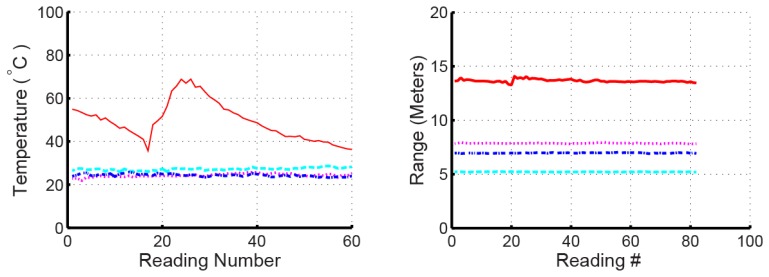
Simultaneous range and temperature measurements made on four 915 MHz OFC sensors in an open atrium environment. The sensors were placed radially around the transceiver at the measured range distances. Three sensors operate at room temperature, while 1 sensor is heated with a heat gun and then allowed to cool in open air.

To demonstrate multi-parameter sensing using the OFC SAW temperature sensors, the range of 4 sensors was extracted from the delay while simultaneously extracting temperature, shown in Figure 18.

These are the identical sensors used in previous experiments and use single-track devices. Temperature sensing is extracted using the adaptive filter approach using the OFC reflector code and range is extracted from delay measurements. For this experiment, the sensors were placed at various positions within a large atrium, with a maximum range of 14 meters. Three of the sensors were at room temperature and one sensor was subjected to heating and cooling cycles using a heat gun. Each sensor was identifiable and temperature was measured to within ±2 °C.

The temperature and range extraction are coupled in the embodiment used due to the single acoustic track, meaning that an error in one variable results is an error in the other. However, the experiment demonstrates the ability of the devices to have multi-variable parameters on a single die.

### e. Magnetic Field Sensor

The previous sensor discussions used the sensing within the piezoelectric die by changes in the material parameters. The SAW device embodiment can also be integrated with external or “off-die” sensors (ODS), where the SAW die is used principally as the communication link to encode the information and re-transmit the signal. One such device form is to use an external sensor and affect the properties of a variable reflector by changing a transducer reflector response under varying load conditions on the SAW die by the ODS impedance change [24,25]. In this case, the device is used principally as the RFID communication link, and the reflected signal from the antenna is modified based on the sensed information.

**Figure 19.**
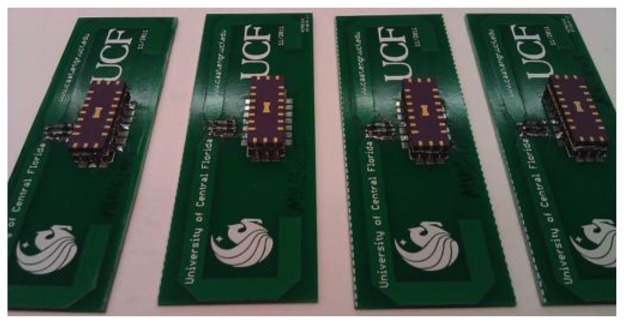
Photo of four packaged 915 MHz OFC closure sensors with PCB antenna. The sensors use miniature REED switches as the magnetic sensing element, seen between the folded dipole antenna and the top of the surface mount package. Open and close setting is determined by a change in the received amplitude of the device. This embodiment demonstrates that the SAW can be used as the communication link when integrated with external “off-board sensors”.

A different approach to be presented is to change the impedance between the SAW die and the antenna using an ODS. An example device is shown in Figure 19 for a closure or magnetic field sensor. The ODS in this example is an off the shelf miniature REED switch that has good RF characteristics at 915 MHz; less than 0.5 dB transmission loss in a 50 ohm system when closed, and more than 15 dB isolation when opened. The REED devices are placed in parallel with a resistor between the SAW surface mount package and each side of the dipole antenna. In the present example, a simple chip resistor of approximately 10 ohms is used which produces an amplitude change in the correlation of approximately 4 dB between “on” *versus* “off” state. The resistor value can be chosen to provide any required attenuation, based on impedances and differential levels desired. In the closed position, the REED switch shorts the resistor and the sensor's loss decreases, indicating closure. This device is actuated by proximity of magnetic fields and can be used for a variety of applications, including a simple wireless security system. For this example, a home security system magnet was used to activate the REED switch. The hardware requires no changes from the previous examples, and simple software changes and a new graphical user interface, allow simultaneous detection of the 4 OFC encoded SAW devices and display of the information. As a side benefit, there are many ODS embodiments that can be envisioned using similar approaches, such as stub tuning, antenna tuning, or resonance tuning. The sensor may also be embedded into the antenna or be an integral part of the antenna construction, such as in a corrosion sensor. The ODS device could also be chosen from any suitable off the shelf parts, such as light, humidity sensors, *etc.*, which makes for rapid wireless sensor development.

## 10. Discussion and Conclusions

Demonstration of several passive wireless SAW sensors and their interrogation results using a coherent correlator transceiver approach have been shown. In particular, the use of OFC SAW devices as the sensor platform for both on-die and off-die SAW sensing have been presented. This paper provides the latest results of a successfully operating OFC SAW sensor system at 915 MHz and various sensors have been simultaneously interrogated and data extracted. This paper verifies the feasibility of the SAW OFC RFID sensor concept and provides, to the authors' knowledge, the greatest range and number of simultaneous operational sensors reported to date. In addition to the examples presented, our group has also demonstrated wireless reversible-hydrogen-gas sensing, liquid level sensing, and cryogenic liquid and temperature sensing. The results demonstrate what has been achieved, but the possibilities go well beyond. The same hardware platform is used for all these differing sensor types, and only the software is reconfigured for parameter extraction. The current emphasis has been on aerospace applications, but the wireless passive SAW RFID and sensor concept will have a wide range of military, industrial and commercial applications. The devices are small, solid state, totally passive with no external power except interrogation energy, are radiation hard, and can be configured for ultra-wide band (UWB) operation. The current demonstrated range has been in the 10's of meters, but predictions indicate that 100's of meters are possible with coherent integration using different hardware configurations. The current system shows the feasibility of the OFC sensor and coherent correlator concepts, and future developments will enhance many of the device and system performance parameters.
